# Co-producing Knowledge on Ecosystem Services: A Multidimensional Participatory Mapping Study in the Kat River Catchment, South Africa

**DOI:** 10.1007/s00267-026-02538-6

**Published:** 2026-06-30

**Authors:** Oghenekaro Nelson Odume, Esther Ahuoiza Seriki, Chenai Murata, Chika Felicitas Nnadozie

**Affiliations:** 1 Institute for Water Research (IWR), Rhodes University, Old Geology Building (Off Artillery Road), P.O Box 94, Makhanda (Grahamstown), 6140 South Africa; 2 Department of African Studies, University of Vienna, Vienna, Austria

**Keywords:** Freshwater systems, Cultural services, Provisioning services, Perceived spatial and temporal ecological changes, Local ecological knowledge

## Abstract

Freshwater ecosystem services in peri-urban catchments are increasingly threatened by land-use intensification, infrastructure deficiencies, and climate variability, with significant implications for environmental management and community well-being. This study examines ecosystem services in the Kat River Catchment, Eastern Cape, South Africa, using a multidimensional participatory mapping approach to assess service types, perceived importance, perceived spatial and temporal changes in availability and quality. A total of 54 stakeholders, representing communities within the catchment (the Kat River Catchment Forum), commercial farmers, subsistence farmers, and non-farming households participated in this study. There were 23 participants in a participatory mapping workshop and 31 semi-structured interview respondents. Provisioning services such as water, reeds, wood, sand, fish, and medicinal plants were consistently rated as highly important to local livelihoods, while cultural services including spiritual and recreational uses were rated as moderately important. Most ecosystem services were perceived to have deteriorated since the year 2000, particularly water quality and availability, reeds used for craft production, and fish for food and recreation. Participants attributed these declines to climate variability, agricultural intensification, failing wastewater treatment infrastructure, and inadequate solid waste management. The findings demonstrate how participatory approaches can generate locally grounded evidence of ecosystem service degradation and identify governance and infrastructure gaps. Integrating local ecological knowledge into catchment management, strengthening wastewater treatment performance, and improving pollution control are critical for enhancing ecosystem service sustainability and supporting adaptive environmental management in peri-urban river systems.

**Figure:**
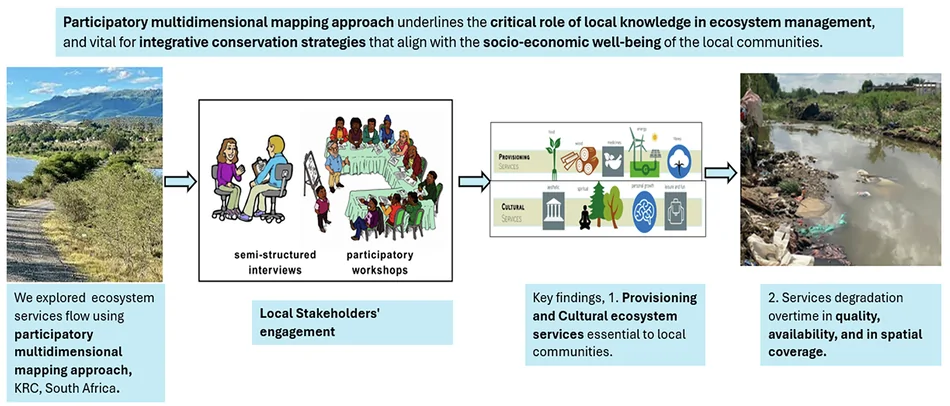

## Highlights

- Participatory mapping identified locally prioritised freshwater ecosystem services in a peri-urban catchment.
- Provisioning services were perceived as essential for livelihoods, with cultural services remaining socially significant.
- Most ecosystem services have declined since 2000, particularly water quality, reeds, and fish.
- Declines were linked to climate variability, agricultural intensification, and wastewater infrastructure failure.
- Integrating local ecological knowledge can inform adaptive catchment and pollution management.

## Introduction

Freshwater ecosystems, including rivers, lakes, and streams, provide a wide range of ecosystem services (ES) such as such as water supply, food provision, materials, cultural identity, that are fundamental to human well-being, and spiritual fulfilment (Allan et al. 2021; Assessment Millennium Ecosystem 2005). Ecosystem services are commonly conceptualised as the direct and indirect benefits that people obtain from ecosystems (Costanza 2008; Daily 1997), and the ES framework has become central to both scientific and policy debates on biodiversity, conservation, and sustainable development (De Groot et al. 2010; Grêt-Regamey et al. 2017). In river catchments, especially in the Global South, ES play a crucial role in sustaining rural and peri-urban livelihoods, yet they are under increasing pressure from land-use change, pollution, water abstraction, and climate variability (Anderson et al. 2019; Mack et al. 2024).

Mapping and assessing ES are now widely recognised as essential for informing policy and management (Maes et al. 2012; Martínez-Harms et al. 2015). Biophysical mapping approaches, which use indicators such as land cover, hydrology, and ecological models, have been extensively developed and applied to quantify and map ES at multiple scales (Dufour et al. 2018; Vihervaara et al. 2018; Zhang et al. 2017). These methods are powerful for revealing spatial patterns and trade-offs in ES supply, but they often under-represent local knowledge, socio-cultural values, and lived experiences of environmental change (Canham et al. 2019; Grêt-Regamey et al. 2017). This limitation is particularly relevant in African catchments, where formal monitoring data are sparse and communities rely heavily on freshwater ES for their livelihoods and cultural practices (Biggs et al. 2017; Adom et al. 2023).

Participatory mapping (PM) and public participation have emerged as important complementary tools for ES assessment, as they explicitly integrate stakeholder perceptions, experiences, and values into spatial-temporal information about ES (Brown and Fagerholm 2015). Recent studies have demonstrated the potential of PM to capture locally important ES, especially cultural and relational values, and to inform planning and decision-making (Fritz et al. 2019; Schwartz et al. 2022; de Sherbinin et al. 2021). However, many participatory ES mapping efforts have focused on single dimensions, such as spatial distribution or importance, without simultaneously addressing temporal change or socio-ecological drivers (Boeraeve et al. 2018). In addition, relatively few studies have applied multidimensional participatory ES mapping in African river catchments, despite evidence of widespread freshwater ecosystem degradation and growing water insecurity (Steyn et al. 2019; Nnadozie and Odume 2019).

The Kat River Catchment (KRC) in South Africa’s Eastern Cape Province provides a pertinent case for examining these issues. Biophysical studies document significant deterioration in river water quality and ecological condition linked to agricultural intensification, failing wastewater treatment works, and land-use change (Soviti 2016; Akamagwuna et al. 2022). Yet, there is limited understanding of how local communities perceive ES, their importance for well-being, and the ways in which these services are changing over space and time. This gap limits the ability of water managers and policymakers to design interventions that align with local priorities and knowledge systems.

To address this gap, the present study applied a multidimensional PM approach grounded in the ecosystem (dis)services flow framework (Odume and de Wet, 2019; Haines-Young and Potschin 2010; Potschin-Young et al. 2018). Specifically, this study aims to (1) identify ES perceived by local communities and stakeholders in the KRC and to assess their perceived importance for well-being; (2) map perceived spatial and temporal changes in these services over the past two decades; and (3) examine the socio-ecological drivers associated with these changes. By integrating spatial, temporal, and socio-ecological dimensions of ES through PM and qualitative interviews, this study contributes to the growing body of work on ES in social-ecological systems. Understanding how ecosystem service degradation is perceived by resource-dependent communities is critical for designing effective, locally responsive environmental management interventions. Also, it provides empirically grounded insights for designing context-sensitive, participatory management strategies in peri-urban freshwater catchments in the region.

## Materials and Methods

### Study Area

**Fig. 1:**
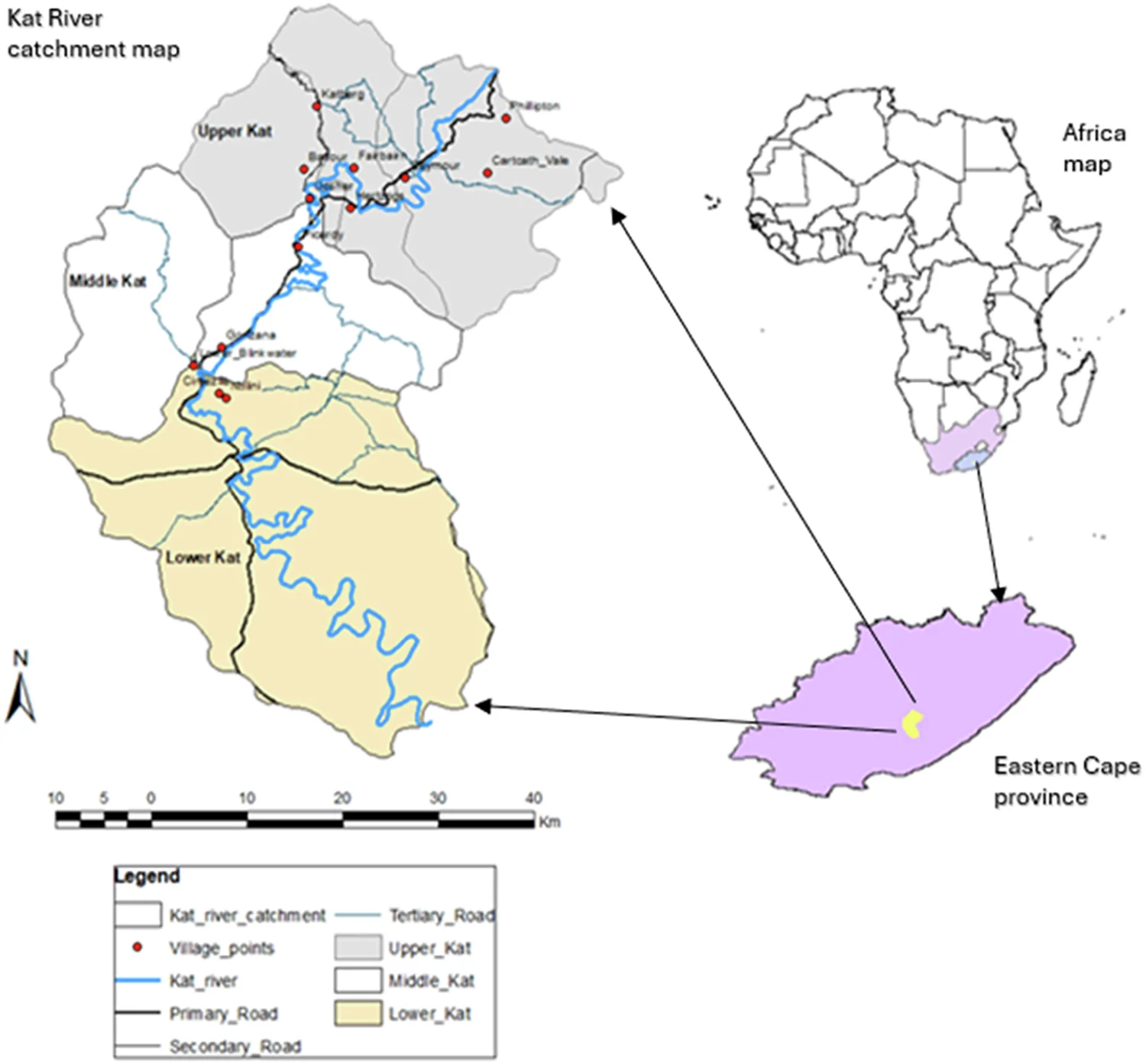
Kat River catchment showing Upper, Middle and Lower Kat (sub catchment) and villages

The KRC is situated in the Amatole District of the Eastern Cape Province, South Africa (SA), and comprises three sub-catchments: Upper Kat, Middle Kat, and Lower Kat (Fig. 1). The catchment covers approximately 1715 km² (Statistics SA 2011). The Kat River originates in the Hogsback and Katberg mountain ranges and provides water for domestic use, irrigation, livestock, and cultural practices (Rowntree and du Preez, 2008). Land use is diverse, including commercial citrus farming, smallholder livestock and crop production, and scattered rural settlements (Motteux and McMaster 2002; Naidoo et al. 2008).

The catchment experiences a temperate climate with seasonally variable rainfall, high evapotranspiration, and recurrent droughts, which together heighten competition for water resources and increase ecological vulnerability (Gush et al. 2002; Steyn et al. 2019). Previous studies have documented agricultural pollution, failing wastewater treatment works, and land-use change as key pressures on freshwater ecosystem condition in the KRC (Soviti 2016; Akamagwuna et al. 2022).

### Research Design and Sampling

A qualitative case study design that combines PM with semi-structured interviews to elicit stakeholder perceptions and lived experiences of ES was adopted. A purposive sampling strategy was used to recruit participants based on their residence in the catchment and direct experience with local water resources and land use. Participants recruitment for the PM workshop and semi-structured interview was aided by the KRC gatekeeper, who serves as the Deputy Chairperson of the Kat River catchment forum (KRCF). The KRCF is a non-statutory forum set up for participatory governance of water resources in the catchment. Its membership is inclusive of all interested and affected parties within and outside of the catchment. The approach to participant recruitment is appropriate for participatory ES research where the objective is to capture diverse and information-rich perspectives rather than to obtain a statistically representative sample (Reed 2008; Brown and Fagerholm 2015).

In total, 54 participants (18 women, 36 men) aged 18-66 years took part in the study (Table 1). They included members of the Kat River Catchment Forum (KRCF), commercial farmers (CF), subsistence farmers (SF), and non-farming households (NFH) from across the Upper, Middle, and Lower Kat sub-catchments.

**Table 1:** Demographic profile of participatory mapping workshop and interview participants

Participatory mapping workshop participants		
Gender	Male	17
	Female	6
Age range (in years)		18- > 66
No. of participants per sub-catchment area	Upper Kat	11
	Middle Kat	8
	Lower Kat	4
Years of living in the catchment (years)	1-5	2
	6 - 15	4
	16 - 25	3
	26 - 35	5
	> 35	9
Employment status	Not employed	17
	Employed	3
	Studying	3
Total no. of participants		**23**
**Semi-structured interview participants**		
Gender	Male	19
	Female	12
Age range (in years)		18 -> 66
No. of participants per sub-catchment area	Upper Kat	16
	Middle Kat	11
	Lower Kat	4
Years of living in the catchment (years)	1-5	1
	6 - 15	5
	16 - 25	2
	26 - 35	5
	> 35	18
Employment status	Not employed	16
	Employed	9
	Student	6
**Total no**. **of participants**		**31**

### Multidimensional participatory mapping of ecosystem services

Participatory mapping was selected because it enables the spatialisation of local ecological knowledge and perceptions that are typically absent from biophysical ecosystem service assessments, particularly in data-scarce settings. A 2-day PM workshop was held with 23 KRCF members, representing a range of water-related interests and land-use practices. The PM workshop was conducted over the period of 30–31 May 2023. The workshop aimed to capture participants’ perceptions of: (i) the ES provided by the catchment; (ii) the importance of these services for their well-being; and (iii) perceived changes in these services over time and space.

Working in facilitated small groups, participants were provided with large-format topographic maps of the catchment. In a first step, together they identified and located key benefits derived from the Kat River and its riparian areas (for example, water for domestic use, irrigation, livestock, reeds, fish, spiritual sites, recreational areas). The identified benefits were classified into ES following the Assessment Millennium Ecosystem (2005). In a second step, participants ranked the importance of each identified service for their households and livelihoods as either very important, moderately important, or not important.

To capture temporal change, participants were asked to reflect on whether the benefits associated with each ES had increased/improved, remained the same, or decreased over three time periods: 0–5 years, 5–15 years, and >15 years. To capture spatial change, participants indicated whether the spatial coverage of each ES had increased, remained unchanged, or decreased within the catchment, and adjusted map annotations accordingly. Notes were taken during group discussions to document explanations and narratives associated with these perceived changes.

### Semi-structured interviews

To complement the workshop and deepen the analysis of socio-ecological dynamics, 31 semi-structured interviews were conducted with additional stakeholders from all three sub-catchments. Interviewees included commercial and subsistence farmers, non-farming households, and individuals involved in water or land management. The interview guide explored (i) personal and household use of ES, (ii) perceived changes in ES quality, availability, and spatial distribution, (iii) perceived drivers of these changes (for example, land-use practices, infrastructure, climate variability), and (iv) impacts on livelihoods and cultural practices. Interviews were conducted in participants’ preferred language with the assistance of a local translator where needed, audio-recorded with consent, and later transcribed verbatim.

### Data analysis

Workshop maps were digitised as points and analysed to identify the location and distribution of perceived ES across the catchment. The analysis was tabulated (Tables 2–4) and interpreted quantitatively. While the primary aim was qualitative, the importance rankings and perceived change categories were also summarised quantitatively (for example, counts or percentages of groups identifying a service as very important or deteriorating). Interview and workshop discussion transcripts were imported into qualitative analysis software NVivo version 14. Nvivo software was used to organised and find initial patterns (codes), and thematic analysis was subsequently applied to interpret and analysed the data. An initial coding framework was developed based on the research questions and the ecosystem (dis)services flow model (Odume and de Wet 2019). This framework was iteratively refined to incorporate inductive codes emerging from the data, including locally salient categories of benefits, pressures, and impacts. To enhance analytical rigour, coding was cross-checked, and discrepancies were discussed until consensus was reached based on the aim of the study. Themes were then synthesised to link perceived ES, their importance, and spatio-temporal changes with underlying socio-ecological dynamics.

**Table 2:** Participants perceived ES benefits, importance, its contribution to well-being and locality, in the KRC

ES	Benefits	Locality	Contribution to well-being	Perceived importance
Water	Provisioning benefits (PB)	Upper Kat (UK) Middle Kat (MK) Lower Kat (LK)	Drinking, domestic use, irrigation & livestock	*Very important*
Sand	PB	UK	Construction	
Wood	PB	MK	Construction	
Fishing	PB	UK, MK, LK	food & recreation	
Reed	PB	UK, MK	Construction	
Medicinal Plants	PB	MK	Food and local herbs	
Spiritual benefits	Cultural benefits (CB)	UK, MK	Religious activities	*Moderately important*
Recreation	CB	UK, MK, LK	Physical & mental

**Table 3:** Perceived changes in temporal dimensions of the ES benefits

Perceived ES	Temporal scale in years	Perceived benefits to society
Water	>15	Deteriorating
Sand	0 - 5	
Wood	5 - 15	Unchanged
Fishing	5 - 15	
Reed	>15	
Medicinal Plants	0 - 5	
Spiritual benefits	0 - 5	
Recreation	>15	

Cultural services showed a more nuanced pattern. While some participants felt that spiritual and religious practices at the river had persisted despite environmental change, others reported reduced use of certain sites due to perceived pollution or physical access constraints. Overall, the perceived temporal trends indicate a general deterioration in key provisioning services and a more complex but still negative pattern for cultural services.

**Table 4:** Perceived changes in spatial dimensions of the ES benefits

ES	Period in years	Perceived spatial coverage over time
Water	>15	Unchanged
Sand	0–5	Decreasing
Wood	5–15	
Fishing	5–15	
Reed	>15	
Medicinal Plants	0–5	
Spiritual benefits	0–5	
Recreation	5–15	

## Results

### Perceived Ecosystem Services, Benefits and Importance

Participants identified a range of ES supplied by the KRC, predominantly provisioning services (water, reeds, wood, sand, fish, medicinal plants) and cultural services (spiritual practices, recreation) (Table 2). Regulating and supporting services such as water purification, nutrient cycling, and flood regulation were not explicitly recognised as ES, even though participants described related processes (for example, “clean” water, flood protection) in their narratives.

From Table 2, the perceived importance of the ES were ranked with numbers (1, 3, 5) and colours (red, orange, green) respectively, where 1 and red indicate *not important*, 3 and orange indicate *moderately important*, and 5 and green indicate *very important*. The participants ranked with numbers while for the purpose of reporting, colours were used instead. The locality represents the sub-catchment location of the perceived ES been assessed. Provisioning services were consistently perceived as very important to household well-being. Eighty percent of 54 participants ranked the provisioning services from the KRC as very important. Water from the river and associated infrastructure was used for drinking, cooking, laundry, brewing traditional beer, livestock watering, and irrigation. Reeds and wood, especially in the Upper and Middle Kat, were used for building materials and craft production (such as, reed mats), contributing both to subsistence and cash income. Sand extracted from the riverbanks was used for house construction and local trade. Fish provided food, income through local sales, and recreational opportunities, thereby serving both provisioning and cultural functions.

These perceptions are illustrated by participant accounts, for example:

*“The water is not safe. We drink the water like that (but) sometimes boil before drinking (NFH).”*

*“Currently we suspect that there are no more fishes in the river because fishermen and the youth no longer hunt for fish, like how they did in the past 5 years (SF)*.”

Cultural services were perceived and ranked by 40 percent of the overall participants as moderately important. The river and specific reaches were identified as sites for communicating with ancestors, initiation into traditional healing, and Christian baptism. Recreational activities, particularly swimming and informal leisure at the river, were also mentioned as important for physical and mental well-being.

### Perceived Temporal Changes in ES Benefits

Participants reported that many of the ES had deteriorated in quality and availability since around the year 2000, with particularly marked declines associated with the 2015-2020 multi-year drought in the Eastern Cape. Water was perceived to have deteriorated in both quality and quantity, affecting its suitability for drinking, domestic use, irrigation, and livestock watering. Interviewees frequently described the water as “not safe” and expressed concern that even boiling did not fully mitigate perceived risks.

Participants perceived all ES to be deteriorating, although the timing and rate of decline varied across services (Table 3). Water quality and availability were consistently described as having worsened over a period exceeding 15 years (>15), indicating a long-term decline in this foundational service. Fishing, reeds, and medicinal plants all showed downward trends, though their temporal patterns differed. Most respondents located the decline in fishing within the past 5–15 years, attributing it to pollution, habitat disturbance, and altered flow conditions. Several interviewees noted that younger people no longer fish as frequently as in the past, suggesting a decline in both the resource and its cultural significance. Reeds were reported to have declined over a period exceeding 15 years (>15), as prolonged low flows and more frequent floods reduced their availability for craft production. Conversely, the reduction of medicinal plants was described as a more recent phenomenon occurring within the past 5 years (0–5). Sand availability was also perceived to have decreased mainly during this recent period, largely due to flood-driven redistribution and riverbank erosion. Cultural services, including spiritual sites and recreation areas, presented a more complex pattern. While some participants noted that key cultural areas remained spatially unchanged, many emphasised that the quality and usability of specific locations (such as swimming spots and baptism sites), had deteriorated, in some cases over periods longer than 15 years (>15).

### Perceived Spatial Changes in ES Benefits

Perceptions of spatial change varied across ES. Participants generally reported that the spatial coverage of water for domestic use and irrigation had remained constant, largely because settlements and irrigation schemes remain tied to fixed infrastructure. However, they noted that the quality of this water had declined, especially downstream of settlements and agricultural areas.

Spatial dimension of water for domestic use and irrigation was generally perceived to have remained spatially unchanged for more than 15 years (>15), largely because households and irrigation systems remain tied to fixed infrastructure, although participants noted that water quality had declined at certain downstream locations (Table 4). While the spatial dimension of reeds and fishing grounds was understood to have shifted over time, reflecting changes in river flow and sediment dynamics. Several groups indicated that reed stands had contracted in particular stretches of the catchment. Recreation areas and some spiritual sites were also perceived to have decreased in spatial availability, especially swimming spots and locations used for baptism or initiation, with participants attributing these reductions to pollution, erosion of riverbanks, and increasing difficulties accessing the river (Table 4). Sand deposits, which are used locally for construction, were described as having decreased in availability during the past five years (0-5), primarily due to flood events that removed or redistributed the deposits on which households depend.

In contrast, the spatial coverage of recreational services (particularly swimming areas) and some spiritual sites were perceived to have decreased. Participants attributed this decline to a combination of lower flows, bank erosion, pollution, and changing social practices. Reeds and fishing grounds were perceived to shift spatially in response to changes in water level, flow, and sediment dynamics. Sand deposits used for construction were reported to be increasingly affected by flood events, which can both remove and redistribute material.

## Discussion

This study employed a multidimensional PM approach to examine how local stakeholders in the KRC perceive ES, their importance for well-being, and their spatio-temporal dynamics. The findings indicate that communities primarily recognise and prioritise provisioning services such as water, reeds, wood, sand, fish, and medicinal plants, while cultural services are perceived as moderately important and regulating and supporting services remain largely implicit. These findings are consistent with studies showing that local communities tend to prioritise tangible provisioning services over more abstract regulating and supporting services, while still attributing significant value to cultural benefits (Reyers et al. 2013; Murata et al. 2019; Brill et al. 2022). Murata et al. (2019) found that rural communities in the Eastern Cape ranked provisioning ES highest, followed by cultural services, with regulatory and supporting services receiving limited recognition. Similar patterns have been documented in other contexts, where communities tend to identify ES that provide direct and tangible benefits (Daw et al. 2011; Mack et al. 2024; Shackleton 2021). The limited recognition of regulating and supporting services can be attributed to their indirect and less visible nature.

The limited recognition of less visible ecosystem functions aligns with earlier work by Lewan and Söderqvist (2002), who showed that even individuals with environmental training more readily recognised visible services like provisioning than regulating services. Their study demonstrated that the ecosystem service concept itself was unfamiliar to many informants, including professionals. This observation resonates with Costanza’s (2008) argument that the ecosystem service framework can be conceptually complex and technically framed in ways that do not easily resonate with non-specialist audiences. However, the present findings do not suggest an absence of ecological knowledge among local communities. Rather, they indicate that ES are perceived through lived experience and practical dependence. Steffen et al. (2015), van Jaarsveld et al. (2005), Anderson et al. (2019), and Murata et al. (2019) describe such knowledge systems as indigenous environmental knowledge, traditional ecological knowledge, or lay ecological knowledge. In this study, these are collectively referred to as local ecological knowledge. Consistent with the Assessment Millennium Ecosystem (2005) and Costanza et al. (2017), communities tend to interpret ES through a utilitarian lens, focusing on direct benefits such as water supply and materials rather than ecological processes such as nutrient cycling or water purification. In this study, local communities tend to value nature based on its immediate usefulnes**s** and tangible benefits rather than its complex biological functions. Importantly, participants demonstrated a clear awareness of long-term ecological change and its implications for household well-being.

The PM process revealed a perceived long-term deterioration in multiple ES, particularly water quality and availability, reeds, and fish. These perceptions align with documented drought events and broader water quality trends in South African rivers (Soviti 2016; Akamagwuna et al. 2022; Steyn et al. 2019). By distinguishing between short-term and long-term temporal scales, participants provided a community-based chronology of ecosystem service change that can complement hydrological monitoring and remote sensing approaches (Gupta et al. 2022). Integrating such locally grounded temporal insights into formal environmental monitoring systems may enhance adaptive management in data-scarce peri-urban contexts.

The findings further highlight the socio-ecological nature of ecosystem service degradation in the KRC. Participants linked ecosystem decline not only to climate variability and drought, but also to agricultural intensification, failing wastewater treatment works, and inadequate solid waste management. These drivers are widely recognised in regional water pollution assessments (Nnadozie and Odume 2019; Wassie 2020). However, biophysical ecosystem service assessments often aggregate such pressures under broad categories such as land-use change or pollution (Dufour et al. 2018), without specifying infrastructure performance or governance gaps. By explicitly identifying wastewater treatment failures and waste disposal practices as perceived drivers, this study adds an institutional and infrastructural dimension to ecosystem service narratives. Such specificity is critical for environmental management because it directs attention toward actionable interventions rather than abstract stressors.

Methodologically, the study demonstrates the utility of PM in capturing locally salient ES in a peri-urban African setting. Similar to findings from Europe and Latin America (Fagerholm and Palomo 2017; Boeraeve et al. 2018; Schwartz et al. 2022), PM was effective in revealing cultural and place-based values that may not emerge from purely biophysical mapping. Nevertheless, the approach has limitations. It relies on perceptions rather than direct ecological measurements, may under-represent less visible regulatory and supporting services (Brown and Fagerholm 2015; Brown and Kyttä 2014), and can be shaped by intra-community power dynamics. In this study, these limitations were partially mitigated through the combination of group mapping and individual interviews, along with iterative and transparent coding procedures. Future research should further integrate participatory approaches with quantitative ecosystem service indicators to strengthen cross-validation.

Spatial perceptions also carry important management implications. These perceptions align with regional evidence that land-use change, altered flow regimes, and sediment dynamics reshape the spatial distribution of ES in South African catchments (Dabrowski and de Klerk 2013). The PM approach used here adds a locally grounded, perception-based dimension to these broader patterns. While certain services such as medicinal plants and water for irrigation were perceived as spatially stable, others, including recreational and religious services, showed spatial contraction. Similar spatial shifts associated with land-use intensification have been documented in the Eastern Cape (Egoh et al. 2012). Cultural services were sometimes perceived as resilient despite environmental degradation, reflecting the strong cultural identity of amaXhosa communities in the region (Murata 2020). Rivers remain sites of baptism, ancestral communication, and traditional healing (Jacobs 2003; Kaschula and Ralarala 2004), although declining water quality has affected such practices elsewhere in South Africa (De Groot et al. 2010; Strang, 2015; Verbuyst 2022). These findings reinforce the importance of participatory approaches in ecosystem management (Reed et al. 2018), as incorporating community perspectives can enhance legitimacy and sustainability of conservation initiatives (Bennett 2014; Reed 2008). Based on the findings, a comprehensive report detailing key recommendations was compiled and presented to members of the KRC forum and representatives from the Department of Water and Sanitation (DWS) to guide subsequent mitigation and implementation strategies.

Overall, this study contributes to the ES literature in three key ways. First, it applies a multidimensional PM framework that simultaneously considers perceived benefits, importance, and spatio-temporal change in a peri-urban African river catchment, a context underrepresented in ecosystem service research (Fritz et al. 2019; de Sherbinin et al. 2021). Second, it demonstrates how community-based temporal classifications can complement hydrological monitoring and remote sensing in adaptive catchment management frameworks (Gupta et al. 2022). Third, it highlights infrastructure and governance failures, such as wastewater treatment and solid waste management deficiencies, as perceived drivers of ecosystem degradation. Together, findings highlight the urgent need to strengthen institutional coordination, wastewater treatment performance monitoring, and enforcement of pollution control regulations in the KRC. Participatory ecosystem service mapping can serve as a diagnostic tool for identifying locally salient environmental risks and governance gaps, supporting adaptive management approaches at catchment scale. In the context of the KRC, participatory mapping can be seen as a useful tool for catchment planning and monitoring, eliciting stakeholder inputs, experiences and perspectives into formal decision-making processes.

## Conclusion

This study combined PM and semi-structured interviews to develop a multidimensional (including spatial, temporal, and socio-ecological) understanding of ES in the KRC. By integrating local ecological knowledge with spatial and temporal analysis, it provides a nuanced account of how communities experience and interpret ecosystem service change. Provisioning services are perceived as vital to livelihoods, while cultural services remain socially important; however, both categories are widely viewed as deteriorating in quality and availability. Regulating and supporting services remain largely implicit in community discourse, despite their ecological importance. The findings underscore the urgency of strengthening adaptive and participatory catchment management strategies. Incorporating local perceptions into ecosystem service assessments can improve alignment between management priorities and community needs. Also, it can enhance institutional legitimacy, and support targeted interventions to address pollution, infrastructure performance, and land-use pressures. Future research should deepen integration between participatory approaches and biophysical ecosystem service indicators, enabling co-production of knowledge that supports resilient freshwater governance in peri-urban contexts. These insights are directly relevant for catchment management authorities seeking to integrate ecosystem service assessments into participatory water governance and restoration planning. Further, this contributes to advancing the understanding of how participatory ecosystem service mapping can inform context-sensitive environmental management in the Global South.

## Data Availability

No datasets were generated or analysed during the current study.
